# Tumor suppressor function of formyl peptide receptor 1 in gastrointestinal cancers: a focus on the underlying signaling

**DOI:** 10.3389/fcell.2025.1733396

**Published:** 2025-12-05

**Authors:** Maria Marotta, Federica Liotti, Rosa Marina Melillo, Nella Prevete

**Affiliations:** 1 Institute of Endotypes in Oncology, Metabolism and Immunology (IEOMI), National Research Council, Naples, Italy; 2 Department of Molecular Medicine and Medical Biotechnology, University of Naples Federico II, Naples, Italy; 3 Department of Translational Medical Sciences, University of Naples Federico II, Naples, Italy

**Keywords:** formyl peptide receptor 1, gastrointestinal epithelia, cancer, tumor suppressor, signaling

## Abstract

The homeostasis of a tissue such the gastrointestinal (GI) tract is of fundamental importance for human health, given its constant exposure to a wide variety of external substances. The Formyl peptide Receptor 1 (FPR1) is an innate immune receptor belonging to the FPR family, that can recognize various endogenous danger- and exogenous pathogen-associated molecules, triggering inflammation. Importantly, depending on the context and on the specific ligand, FPR1 can also stimulate inflammation resolution. Thus, FPR1 is a critical actor in GI physiopathology. Interestingly, not only FPR1 participates and is necessary for maintaining homeostasis, but it also exerts strong tumor suppressor properties in this tissue. The present review discusses the mechanisms responsible for this specific function of FPR1 in cancer of the GI tract, focusing in particular on FPR1-mediated signal transduction.

## 1 Introduction

The gastrointestinal (GI) epithelium has an active role in forming a selective physical and biochemical barrier between luminal content and the underlying tissue compartment. It creates and orchestrates a complex and balanced network regulating microbiota and mucosal immune system, thus ensuring tissue homeostasis maintenance. However, alteration of microbiota, infection, mechanical or pathological injury or intrinsic defects of the epithelial intestinal barrier can often cause the loss of homeostasis and allow the establishment of a chronic inflammatory state (Serhan et al., 2015; Prevete et al., 2017; Fishbein et al., 2021; Liotti et al., 2022a; Liotti et al., 2022b).

In physiological conditions, after an insult that causes epithelial wound, an acute inflammatory response occurs, characterized by pro-inflammatory mediators (e.g., eicosanoids and cytokines) production and innate immune cells’ recruitment (Gilroy, 2021; Roviello et al., 2022). After that, inflammatory response is dampened by inflammation resolution process, better known as restitution phase, induced by different actively produced pro-resolving mediators (Gilroy, 2021; Roviello et al., 2022).

Defects in restitution phase can lead to an exacerbated non-resolving inflammation, that could initiate or sustain the progression of numerous inflammation-driven diseases, including cancer (Prevete et al., 2018b).

Among mediators that contribute to the resolution response, there are lipidic molecules (Sugimoto et al., 2016), known as Specialized Pro-resolving Mediators [lipoxins (LX), resolvins (Rv), maresins (MaR) and protectins (PD)] (Serhan and Savill, 2005; Liotti et al., 2022a); proteic mediators such as Annexin A1 (AnxA1), adrenocorticotropic hormone, chemerin peptides, and galectin-1 (Perretti and D’Acquisto, 2009; Leoni et al., 2013; Sugimoto et al., 2016; Prevete et al., 2018b) and gaseous mediators (nitric oxide, hydrogen sulfide, and carbon monoxide) as well as neuromodulators such as acetylcholine (Mirakaj et al., 2014).

Due to this intimate relation between inflammation balance and GI cancer initiation and/or progression, a critical role in this tissue is ensured by innate immune receptors, whose primary role is to sense both exogenous and endogenous “danger” signals and sustain the barrier functions of the epithelia maintaining an equilibrium state (Pott and Hornef, 2012). Indeed, intestinal and gastric epithelial cells express significant levels of innate immune transmembrane or intracellular pattern recognition receptors (PRR), including Toll-like receptors (TLRs), nucleotide-binding oligomerization domain (NOD)-like receptors (NLRs), and formyl peptide receptors (FPRs) (Hooper et al., 1998; Prevete et al., 2018a; Burgueño and Abreu, 2020; Guo et al., 2020; Eshleman and Alenghat, 2021). Accordingly, these PRRs not only play a pivotal role in detecting pathogenic threats and consequently triggering inflammation but are also involved in wound healing and epithelial repair, particularly in tissues constantly exposed to the external environment, such as the GI tract. These receptors contribute to modulate both inflammatory and pro-resolving responses, allowing the immune system to effectively discriminate between damaging agents and tolerated antigens (Pott and Hornef, 2012).

## 2 Formyl peptide receptors and their role in the physiopathology of the GI tract

Human formyl peptide receptors (FPRs), FPR1, FPR2 and FPR3 (Prossnitz and Ye, 1997), are PRRs belonging to the seven transmembrane domain G_i_-protein-coupled receptor (GPCR) family (Simon et al., 1991). They were originally characterized in phagocytes: upon stimulation by microbe- or mitochondria-derived formylated peptides, they sustain chemotaxis and reactive oxygen species (ROS) generation thus triggering inflammation and immune responses (Prossnitz and Ye, 1997; de Paulis et al., 2009). FPRs are promiscuous sensors that can pick up a wide range of exogenous and endogenous agonists, including non formylated peptides, lipids, and host derived danger signals (Migeotte et al., 2006; Ye et al., 2009).

Besides their expression in immune cells, FPRs are expressed also in several epithelia, including the GI mucosa (Le et al., 2002; Prevete et al., 2015a), where they are closely exposed to gut luminal contents (Babbin et al., 2007) to detect exogenous and host-derived ligands, including microbial formyl peptides from certain bacteria species (de Paulis et al., 2009; Wentworth et al., 2010). Beyond simple pathogen recognition, in intestinal epithelial cells FPRs serve also to distinguish pathogenic or commensal bacteria species and as receptors for endogenous pro-resolving mediators such as annexin A1 (AnxA1), lipoxin A4 (LXA4), and resolvin D1 (RvD1), thus triggering inflammation resolution and contributing to wound restitution and gut homeostasis (Babbin et al., 2007; Dufton and Perretti, 2010; Norling et al., 2012; Leoni et al., 2013; Prevete et al., 2015b).

Over decades FPR role in gastrointestinal epithelia has been deepened consolidating the evidence that the presence of this family of innate immune receptors plays a key role in the barrier function maintenance by regulating wound healing (Prevete et al., 2018a). For instance, it has been demonstrated that AnxA1 requires epithelial FPR1 and the enterocyte NADPH oxidase, NOX1, to promote intestinal mucosal wound healing after insult in a ROS-dependent modality (Leoni et al., 2013). FPR1-dependent signaling is necessary to epithelial repair responses by sensing also specific commensal species (i.e., *Akkermansia muciniphila* or *Lactobacillus rhamnosus*) (Babbin et al., 2007; Wentworth et al., 2010; Jeong and Bae, 2020; Liotti et al., 2022b). This process implicates the recruitment of protective microbial species to the site of injury (i.e., the anaerobic bacteria *Akkermansia muciniphila*), and the following activation of FPR1-dependent ROS-mediated wound healing pathways (Alam et al., 2016). Thus, FPRs regulate the enrichment of commensal species in the regions to be repaired and, once activated by these, trigger a wound healing response. Consistently, different studies demonstrate that FPR1^−/−^ or FPR2^−/−^ mice fail to properly heal colon mucosa after damage in chemically induced colitis model and show low colonic crypt length after recovery (Chen et al., 2013; Alam et al., 2014; Jeong and Bae, 2020).

Similarly, *Helicobacter pylori*-derived Hp(2–20) peptide, through the interaction with FPR2 and FPR3, regulates gastric mucosal healing after damage by facilitating epithelial cell migration, proliferation, and neoangiogenesis (de Paulis et al., 2009). Additionally, administration of this peptide has been reported to accelerate colitis recovery in a rat model (Gravina et al., 2018).

These experimental evidence on the key role of FPRs in managing gut homeostasis could explain the evidence in clinical practice of the increased expression of the receptors in inflammatory bowel diseases (IBD), supporting the idea of a compensatory response following inflammation. Crohn’s disease patients show a high expression level of FPR1 in neutrophils (Anton et al., 1989); moreover, ulcerative colitis (UC) patients display an exacerbate activation of FPR1 in their intestines (Leoni et al., 2015). Similarly, UC patients display increased FPR2 expression levels compared to the healthy controls (Vong et al., 2012).

## 3 Functional role of FPR1 in cancer of GI tract

The FPR role in the homeostasis of gastrointestinal epithelia strongly suggests that FPRs may also be involved in the genesis and the progression of cancer. GI carcinomas are typically associated with both sterile and pathogen-induced inflammation. Indeed, an unsolved alteration of gut homeostasis, with prolonged inflammation, sustains inflammatory-based diseases and is strongly associated with the pathogenesis of cancer (Balkwill et al., 2012).

The evidence for the association between FPR1 and gastric cancer (GC) can be divided into two types of contrasting observations: i) FPR1 was detected in human GC specimens, and its levels are correlated with more aggressive clinical parameters and poorer outcome of patients (Cheng et al., 2014); ii) conversely, another study reports a positive association between a specific FPR1 polymorphism, which reduces receptor activity, and the increased risk of human GC (Otani et al., 2011). The first report suggests a positive correlation between FPR1 and GC progression, while the second a tumor suppressor role of the receptor in GC.

By studying the functional activity of FPR1 in GC experimental models, we described that the genetic ablation of FPR1 in human GC cells expressing high levels of the receptor increased their angiogenic and tumorigenic potential *in vivo*; accordingly, enforced expression of FPR1 in GC cells expressing low FPR1 levels caused the opposite effect, drastically impairing angiogenesis and tumor growth *in vivo* (Prevete et al., 2015b). By investigating the molecular mechanisms responsible for these FPR1 activities, we found that FPR1 blockade or silencing caused a drop in the production of endogenous levels of the Specialized Pro-resolving Mediators (SPMs), due to a reduction in their biosynthetic enzymes (i.e., ALOX5 and 15), and their relative receptors (i.e., GPR32, ChemR23, BLT1). A concomitant increase in the angiogenic potential (production of VEGFs, angiopoietin 1 and CXCL1) of FPR1-depleted GC cells could be observed. Consistently, we found that SPMs control the production of angiogenic mediators, since the exogenous administration of SPMs (RvD1 or LXB4) to FPR1-depleted GC cells could suppress their angiogenic potential. Moreover, the blockade of ALOX15, necessary for SPMs synthesis, or of the pro-resolving receptor GPR32, receptor for the SPM RvD1, enhanced angiogenesis and tumorigenic activity of GC cells, mimicking FPR1 depletion. Thus, we demonstrated that GC cells, similarly to many other cancer cells, are endowed with an intrinsic angiogenic potential, that could be negatively controlled by SPMs. These, in turn, are positively controlled by FPR1 (Prevete et al., 2015a; 2017). These data highlight the tumor suppressor function of FPR1 and suggest that the increased expression of the receptor in human GC samples could be related to a compensatory response.

Since SPMs are metabolite of ω3 and ω6 polyunsaturated fatty acids (PUFA), we were able to demonstrate that diet enriched in PUFA could revert the increased angiogenic potential of FPR1-depleted tumors by enforcing SPMs production and counterbalancing the lack of resolution of cancer cells (Prevete et al., 2017). This opens new possibilities to be exploited for gastric cancer treatment and prevention.

Strong epidemiologic evidence showed that loss-of-function polymorphisms of FPR1 are positively associated with poor responses to chemotherapeutic drugs and an earlier mean age of cancer diagnosis (Sztupinszki et al., 2021) also in CRC patients. From a mechanistic point of view, FPR1 exerts a significant anti-cancer effect in CRC models through mechanisms similar to those observed in GC. Indeed, FPR1 activation by its natural ligands (the formylated peptide fMLF) or by commensal bacterial supernatants (*Lactobacillus rhamnosus* GG - LGG), but not by other lactic acid or non-probiotic bacteria (i.e., *Bifidobacterium bifidum* or *Escherichia coli*), sustains a pro-resolving response and dampens angiogenic potential in CRC cells (Liotti et al., 2022b).

Data produced by our laboratory are in line with the results of Chen *and coll.*, who investigated the role of FPRs in colonic epithelial homeostasis, inflammation, and tumor formation in mice deficient for *mFPR1* and/or *mFPR2*. They demonstrated that deletion of both receptors, with a prominent role of FPR2 on FPR1 in mice, increased colon tumorigenesis upon exposure to azoxymethane (AOM) and dextran sulphate (DSS), an inflammatory-driven model of CRC (Chen et al., 2013). Interestingly, this effect could be observed also upon conditional deletion of the receptors only in the epithelial compartment (Chen et al., 2013), thus indicating that the expression of the receptors in the intestinal epithelial cells exerts a critical impact on CRC progression. Accordingly, we demonstrated that FPR1 inhibition in human CRC cells in culture increased the intrinsic angiogenic potential of cancer cells. Moreover, conditioned media from FPR1-depleted CRC cells significantly increased endothelial cell migration and proliferation compared with parental cells (Liotti et al., 2022b). Furthermore, FPR1 deletion in CRC cells strongly impaired tumor-specific immune response, by reducing the ability of Dendritic cells (DCs) to migrate toward dying CRC cells (Le Naour et al., 2023), thus favoring colonic reactive hyperplasia and inflammation-induced colon tumorigenesis (Vacchelli et al., 2015; Vacchelli et al., 2016; Sztupinszki et al., 2021; Le Naour et al., 2023).

We hypothesize that the tumor suppressor role of FPR1 in GI tract is linked to the crucial homeostatic role of this innate immune receptor in such district, that is massively exposed to exogenous and host-derived stimuli and continuously involved in the balance between inflammation and its resolution. This could explain why FPR1, in other cancer contexts, exerts different functions: in the context of human glioblastoma, FPR1 confers a more invasive and angiogenic phenotype and modulates the tumor microenvironment to favor immunosuppression (Huang et al., 2010; Zheng et al., 2023).

## 4 Anti-tumor FPR1 signaling in intestinal epithelium

The above data consolidated the tumor suppressive role of FPR1 in gastrointestinal epithelia (Prevete et al., 2018a). However, the underlying signaling pathways involved in these activities were still undefined.

Classically, in immune cells FPRs mediate cell activation and inflammation responses mainly by evoking the activation of the RAS - MAPK cascade (Zhu et al., 2021), and by stimulating phospholipase Cβ to induce the release intracellular calcium from the endoplasmic reticulum, and subsequently PKC activation and ROS production (Kwan et al., 2008). The production of ROS is dependent on Rac2-mediated NADPH oxidase 2 (NOX2) activation and is functional to microbicidal activity (Bylund et al., 2003).

**FIGURE 1:**
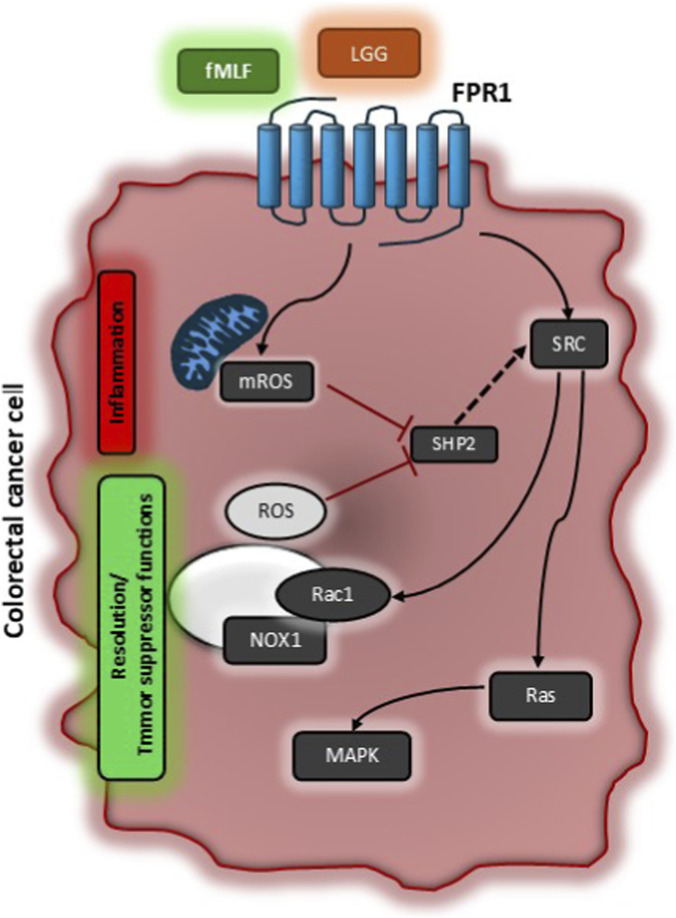
Schematic representation of signaling pathways activated by Formyl Peptide Receptor 1 to exert its tumor suppressor function. In colorectal carcinoma cells, activation of FPR1 by formylated peptides (e.g., fMLF) or bacterial supernatants (e.g., LGG – *Lactobacillus Rhamnosus*) induces a first rapid mitochondrial ROS production (mROS) which is responsible for SHP2 phosphatase inhibition. SHP2 inhibition allows SRC activation which controls the activity of Rac1 and RAS. Rac1 completes NOX1 multicomplex activation sustaining a second wave of NOX1-dependent ROS that reinforce this signaling cycle. RAS classically aids MAPK signaling. The first rapid ROS production by mitochondria is responsible for inflammatory responses (i.e., COX2 expression and PGD2 production), while NOX1-dependent ROS are associated to resolution responses (i.e., ALOXs induction and SPM release) and thus for tumor suppressor properties of FPR1.

We asked which signal transduction pathways downstream FPR1 were involved in FPR1-induced pro-resolving and anti-angiogenic response in GI cancer cells (Liotti et al., 2022b; Liotti et al., 2025). For the first time, we described a mechanism of transduction involving ROS as signaling molecules (Figure 1). We found that the formylated peptide fMLF, by binding FPR1, induces two time- and source-distinguishable waves of ROS production in CRC cells: a first rapid mitochondrial-derived ROS production (mROS), followed by a second late wave, dependent from the first, due to the activation of NADPH oxidase 1 (NOX1). mROS triggers SHP2 phosphatase inactivation, which in turn allows SRC activation, to which follows the activation of the two small GTPases RAS and Rac1. RAS activates MAPK signaling, while Rac1 supports NOX1 activation, that generates the second wave of ROS, reinforcing this signaling cycle (Liotti et al., 2025). By using different approaches and functional assays we showed that mROS production precedes and is necessary for pro-inflammatory mediators’ release, while NOX1-generated ROS are only required for pro-resolving mediators’ synthesis. Finally, we could show that this signaling cascade is essential for the pro-resolving and anti-angiogenic properties of FPR1 in CRC (Liotti et al., 2025) (Figure 1). Importantly, our findings highlight a novel regulatory mechanism in CRC, where both the intensity and duration of ROS signaling critically determine the switch between pro-inflammatory and pro-resolving responses (Figure 1). We propose that the combined action of mROS and NOX1-derived ROS establishes a threshold level of MAPK activation required for the transcription of resolution genes and production of SPMs.

It is consolidated in the literature that, for the switch from inflammation to resolution, it is essential to reach a “threshold” of inflammatory signals (Serhan, 2014; Sugimoto et al., 2016). Our results suggest that the amplitude and temporal dynamics of MAPK signaling could be the key to govern these two distinct transcriptional programs, ultimately dictating whether the cellular response favors inflammation or resolution.

The description of this signaling travels in parallel to evidence describing that the production of ROS is crucial also for the physiologic homeostatic functional role of FPR1 in intestinal epithelial cells (Hurd et al., 2012). It has been reported that FPR1, by binding the cleavage product of the pro-resolving ligand AnxA1 (i.e., Ac2-26 peptide), induces a NOX1-mediated ROS generation able to recover wounds. Specifically, Ac2-26 bond to FPR1 triggers SRC activation, followed by the association of p120 catenin with active Rac1, an essential regulator of NOX1 (Cheng et al., 2006). Then, NOX1-mediated ROS generation inactivates, by oxidation of the cysteine in their active site (Van Montfort et al., 2003), the regulatory phosphatases PTEN and PTP-PEST, with consequent activation of focal adhesion kinase (FAK) (Swanson et al., 2011) and paxillin to promote cell migration and thus mucosal wound restitution (Leoni et al., 2013). Complementary *in vivo* studies using intestinal epithelial cell specific Nox1^−/−^ and AnxA1^−/−^ mice demonstrated defects in intestinal mucosal wound repair (Leoni et al., 2013). Accordingly, systemic administration of AnxA1 prompts wound recovery in a NOX1-dependent fashion (Leoni et al., 2013). Besides AnxA1, *in vivo* studies demonstrated that in colonic enterocytes, FPR1 mediates commensal bacteria-stimulated NOX1-dependent ROS generation, which in turn activates phosphorylation of FAK and ERK, resulting in migration and proliferation that are required for intestinal homeostasis and wound restitution in colonic enterocytes (Alam et al., 2014). Moreover, fMLF - FPR1 interaction induces the activation of Phosphatidylinositol 3-Kinase (PI3K), that in turn activates Rac1 and Cdc42, which are crucial players in intestinal epithelial cell migration and restitution (Babbin et al., 2007). Again, commensal *Lactobacillus rhamnosus* bacterial products, by interacting with FPR1, induce generation of ROS that inactivates DUSP3 phosphatase, thus relieving ERK activation and promoting homeostatic signaling pathway in the mammalian intestine (Wentworth et al., 2011).

Independently from the cells, the stimulation of FPR1 triggers NOX activation thus increasing ROS production. It seems that the ROS produced in immune cells upon FPR stimulation, are fundamental in eliciting innate immune bactericidal functions, while, rather than detrimental, ROS produced through FPR1 stimulation in epithelial cells, serve as protective signaling molecules to convey cell responses to a homeostatic state.

## 5 Discussion

The knowledge of FPR1 signaling responsible for its anticancer properties opens the way for new therapeutic opportunities. Targeting FPR1 or its downstream signaling pathways is a possible option to treat patients with GI cancers. One of the most significant observations in this context is the identification of SHP2 phosphatase inhibition as an essential step for FPR1 anti-angiogenic properties in GI cancer cells. Interestingly, SHP2 phosphatase, with different mechanisms, functions as an oncogene in many other tumor types (Hakak et al., 2000; Agazie et al., 2003; Marotta et al., 2025) and several pharmacologic inhibitors are already available in clinical practice (Ahmed et al., 2019; Drilon et al., 2023; Marotta et al., 2025). Obviously, targeting FPR1 with agonists that induce its pro-resolving, anti-angiogenic and tumor suppressive activities is another possible strategy. The strong preclinical evidence in various disease models, including cancer, suggest that FPR1 agonism is a promising therapeutic approach. Small FPR1 agonists have been developed, but no compounds have still advanced to clinical trials in humans (Yi et al., 2024; Dahlgren and Forsman, 2025). For these reasons it would be desirable to continue the development of the compounds already identified, and to pursue the development of new compounds that are selective for FPR1 and able to activate the correct signaling pathways.
